# Characteristics and Dynamics of Smile in Patients With Skeletal Class II Malocclusion Versus Class I Malocclusion Using Still Digital Video Captures: A Three-Group, Cross-Sectional, Comparative Study

**DOI:** 10.7759/cureus.30704

**Published:** 2022-10-26

**Authors:** Rahaf Maged Kabalan, Reham Khaled Tayyar, Tarek Z Khattab, Mohammad Y Hajeer

**Affiliations:** 1 Department of Orthodontics, University of Hamah Faculty of Dentistry, Hamah, SYR; 2 Department of Orthodontics, University of Damascus Faculty of Dentistry, Damascus, SYR

**Keywords:** interlabial gap, video recording, still images, skeletal class ii division 1 malocclusion, skeletal class ii division 2 malocclusion, class ii malocclusion, nasolabial angle, smile arc, smile position, dynamic smile

## Abstract

Background

Smiling is one of the effective ways for people to express their feelings. It is an integral part of the diagnosis and planning and a key point of the treatment objectives in orthodontic care. Many factors are associated with a pleasant smile, such as correct anatomy, gingival health, and teeth proportion. Therefore, different malocclusion classes can affect the characteristics of smile esthetics. This study aimed to evaluate the effect of skeletal class II malocclusion on the characteristics and dynamics of the smile in the sagittal and frontal planes.

Methodology

The study sample included 60 patients comprising three groups of malocclusion classes, namely, Class I, Class II division 1, and Class II division 2. A video recording was taken for 5-10 seconds for each patient using a specific camera mounted at a fixed distance from the imaged face. Two facial expressions were captured for each patient, one representing the lips at rest and the second representing the unrestricted natural smile. The facial still images were derived from the streaming video recording, and two images were chosen for each plane (the frontal plane and the sagittal plane) for each patient. In total, 12 variables were assessed on these captured images. One-way analysis of variance (ANOVA) was used to detect significant differences between the three groups.

Results

There were statistically significant differences in some of the measured variables. The mean values of thickness of the upper lip, commissure height, gum width, maxillary incisor display, and interlabial gap were greater in the Class II division 1 group than in the other two groups. The proclined incisors were evident in the Class II division 1 group, while the retroclined incisors were evident in the Class II division 2 group.

Conclusions

The skeletal Class II malocclusion influences the characteristics of the smile, either assessed on the anterior or lateral imaging angles, in addition to its influence on the resting position of the lips. Orthodontists should always analyze patients’ facial expressions, including those related to the upper and lower lips at rest and when patients smile naturally. Depending on the results of this analysis, treatment planning could be built to improve the characteristics of the natural smile in patients with Class I and Class II malocclusions.

## Introduction

A smile is the most complex facial expression formed through the synergistic action of expressive facial muscles [1]. It is one of the most important facial functions and is often a measure of success or failure, especially from the patient’s point of view [2,3]. It is considered one of the most important facial expressions to express joy, happiness, mood, and gratitude [4]. The attraction of a smile is an important topic in orthodontics and is often the biggest motivating factor in career improvement and dental health [5,6] as it is considered one of the most frequent demands for orthodontic treatment to obtain the best aesthetic appearance to overcome the psychological and social problems caused by dental abnormalities [7]. There are two main types of smiles, namely, a social smile and an emotional smile, depending on the appearance of the anatomical elements of the smile [8]. The social smile is a repeatable volunteer smile that a person uses in social environments. When taking a picture or meeting someone, your smile indicates that you are friendly and “happy to meet.” It is usually used as a salutation, unrestricted, and stable, and occurs due to mild shrinkage of the perioral facial muscles, and the gums may occasionally appear [8]. While the emotional smile is an involuntary, spontaneous smile is caused by emotional factors such as happiness and has many descriptions, such as laughter, crying, knowledge, or lack of interest, and is controlled by muscles of facial expression [8,9].

Smile registration is taken in static and dynamic conditions, and although these two conditions are separate, they are considered to be interlinked [10]. Recent technological developments have contributed to the study of the smile by looking at it in a video, where the most unified smile (the greatest width) can be identified, thus reducing the error when studying a single shot [10,11]. In the past decade, orthodontists have shown a marked tendency to treat their patients with an emphasis on improving the aesthetics of their smiles [12]. Dong et al. evaluated the aesthetics of the static smile by taking photographs in patients with Class I malocclusion [13]. On the other hand, several studies have analyzed smiles dynamically using digital captures, such as Grover et al. who evaluated incisal display, interlabial gap, lower lip-to-incisal edge distance, upper vertical lip length, occlusal plane angle, and posterior corridors in patients with dental and skeletal Class I malocclusion with different facial patterns [14].

Reviewing the literature reveals that many studies have evaluated the dynamics of smile esthetics in several dental and skeletal malocclusions [11,14]. However, the published studies have not compared these characteristics between malocclusion classes. Therefore, the objective of this study was to compare the effect of skeletal Class II malocclusion (with its two divisions) on the characteristics and dynamics of the smile in comparison with skeletal Class I malocclusion using still images acquired from video recordings.

## Materials and methods

Study design and setting

This was an observational, cross-sectional study for descriptive and analytical purposes conducted at the Department of Orthodontics at the University of Hamah Dental School. The Local Research Ethics Committee Approval was obtained (UHDS-265-04092019/SRC-2373) before the commencement of the study, which was funded by the University of Hamah Postgraduate Research Budget (reference number: 21485110377DEN).

Estimation of the sample size

The sample size was calculated using Minitab® 17 software (Minitab Inc., State College, PA, USA). The intended test was a one-way analysis of variance (ANOVA). With an alpha level of 0.05 and a power of 90%, the smallest clinically significant difference requiring detection in the maxillary incisors display was assumed to be 1.5 mm with a standard deviation of 1.21 mm (from a previous study [15]), and a standardized effect size of 1.23; therefore, a sample size of 18 patients was required for each group. Hence, we decided to include 20 patients in each group, with a total number of 60 patients.

Study sampling and patient recruitment

In this cross-sectional study, the sample comprised 60 patients divided into three groups of equal numbers. This distribution was obtained by applying disproportionate multi-stratified random sampling concerning the skeletal malocclusion class. Our sampling frame was based on checking 800 records of patients who visited the Department of Orthodontics at the University of Hamah Dental School, Hamah, Syria (from December 2019 to June 2020).

**Figure 1:**
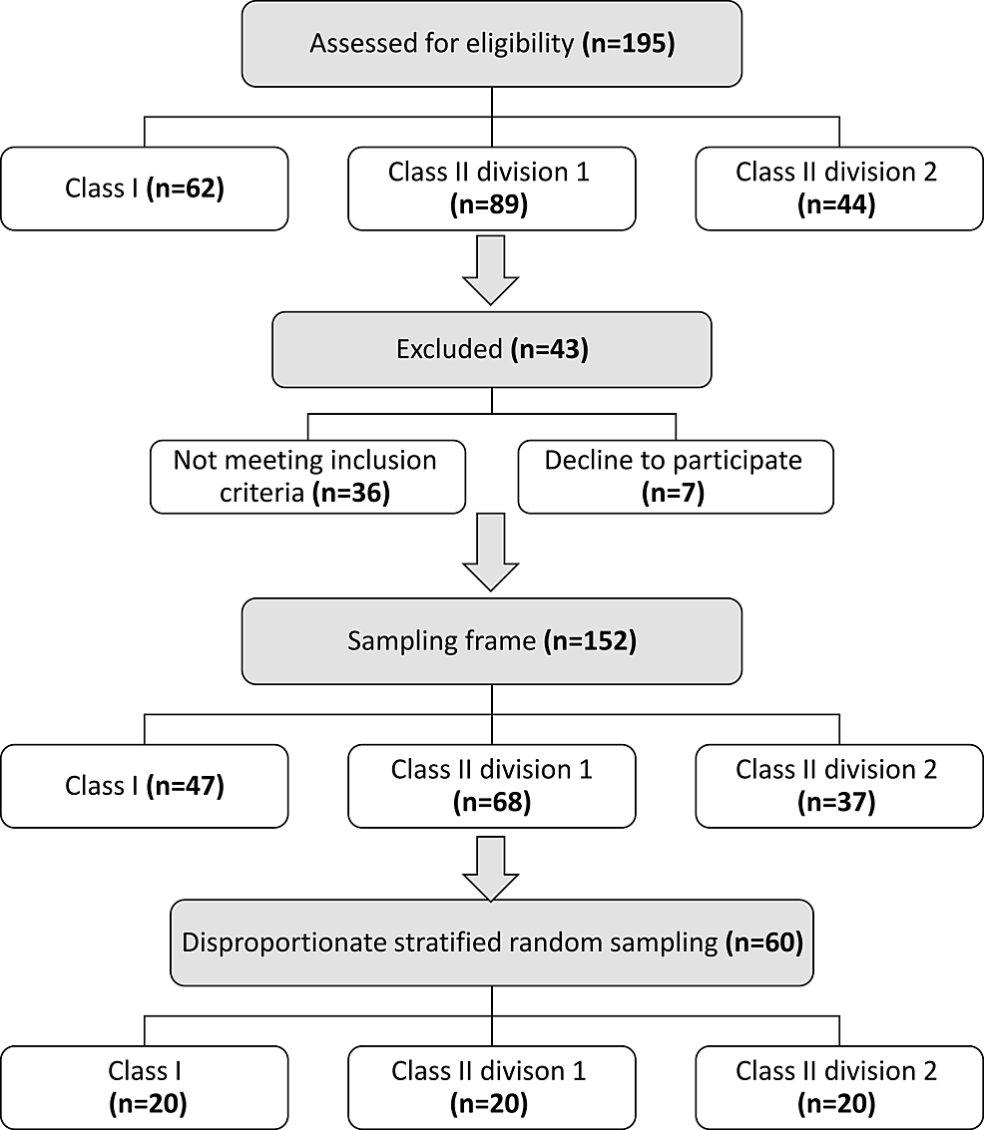
Flow diagram of patients’ recruitment and distribution into the three groups.

After clinical and radiological examination, 195 patients (62 Class I patients, 89 Class II division 1 patients, and 44 Class II division 2 patients) were found suitable for inclusion in the study. In total, 152 patients fulfilled the inclusion criteria (47 Class I malocclusion, 68 Class II division 1 malocclusion, 37 Class II division 2 malocclusion) and were willing to participate and were considered the sampling frame. A total of 60 patients (33 males, 27 females, 20 in each group) were selected and included in the study. Random selection was based on a computer-generated list of random numbers from the sampling frame using disproportionate stratified random sampling. A flow diagram showing patients’ recruitment and distribution into the three groups is presented in Figure 1.

The inclusion criteria were age range of 18 to 28 years, skeletal Class I or Class II malocclusion, presence of all permanent teeth except third molars, symmetrical face with no history of trauma, no previous orthodontic treatment, and no cleft lip or palate. The exclusion criteria were Class III molar or canine relationship, mixed dentition, absent teeth, and dentofacial deformities.

Imaging apparatus and patient orientation

The video was recorded using a digital camera (Digital Camera, NIKON® D3300, Bangkok, Thailand). The method described by Sarver and Ackerman [10] was employed in this study. Patients were instructed to hold their heads in a natural head position [16]. The camera was mounted on a tripod and approximately 4 feet from the patient’s face in the photographic room which provided natural light, so the LED ring light and other lighting equipment were not needed. The camera lens was about 24.2 megapixels and was adjusted parallel to the apparent occlusal plane using digital zoom and focused only on the dentofacial complex of the face in the natural head position (corresponding to the area from the nose to the chin). The patient was asked to smile several times to exercise. Then, two rulers were made to fit perpendicular to help minimize any error resulting from the patient’s head movement. The patient was asked to place them in the pictured area and to fix the horizontal ruler on the chin so that it is parallel to the line passing through the pupils of the eyes in the frontal view.

Capturing procedures

After confirming the position of the rulers and camera, the patient was asked to lick his lips to obtain a resting position and then to say cheese. Filming began with the patient in the resting position of the lips, and the recording continued for one to three seconds before moving to the smiling pose. Each video clip was reviewed, and the frame best represented the patient’s natural, unstrained social smile was selected. The videos were transferred to a desktop computer and were cut using DVD Video Soft Free Studio® 2006 (Digital Wave Ltd, London, United Kingdom) to a set of JPEG images at a rate of 30 photos/second. Subsequently, the appropriate pictures were chosen for the study. The first image represented the rest position (relaxed lips) [17]. The second picture represented the natural, unconventional smile, in which the width between the two commissures was as wide as possible [11].

Two pictures were obtained for each plane so that each patient has four pictures, two of them in the frontal plane (one in the rest position and the other in the natural smile position) and two in the sagittal plane (one in the rest position and the other in the natural smile position).

Outcome measures

**Figure 2:**
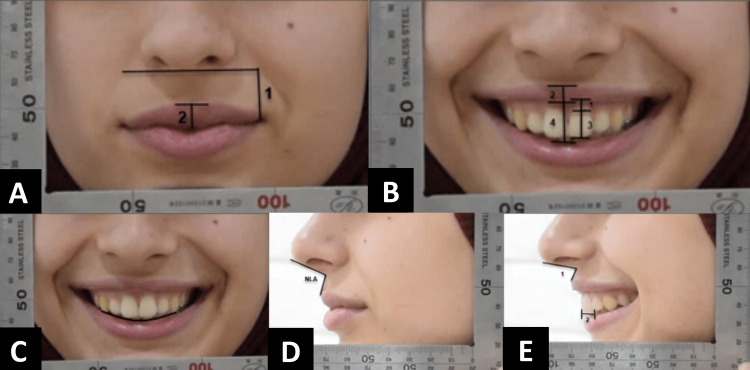
Measurements made on the captured images. (A) 1: the height of the commissure, 2: the thickness of the upper lip; (B) 1: the gum width, 2: the thickness of the upper lip, 3: maxillary incisor display, 4: interlabial gap; (C) smile arc; (D) nasolabial angle. (E) 1: nasolabial angle, 2: bucco-lingual positioning of the upper incisor.

Measurements were made on the selected images in the anterior and lateral filming positions (both the rest and smile facial expressions), and their definitions were taken from previous studies [18-20]. These measurements are explained in Table 1 and illustrated in Figure 2.

**Table 1:** Measurements made on the captured images. *Definitions are taken from McNamara et al. [18]; ^†^Definitions are taken from Nanda [19]; ^††^Definitions are taken from Choi et al. [20].

Measurement	Filming position	Facial expression	Definition
Height of the commissure*	Anterior	Rest	The vertical distance between the commissure and the horizontal line that passes through the base of the nose
Thickness of the upper lip*	Anterior	Rest	The vertical distance from the top edge of the upper lip to the lowest point on the bottom edge of the upper lip
Lower lip to maxillary incisor*	Anterior	Smile	The vertical distance from the deepest point on the middle line at the edge of the lower lip to the cutting edge of the maxillary incisor
Smile arc†	Anterior	Smile	The relationship between the curvature of the incisor edges of the upper incisors and the canines with the curvature of the lower lip (reverse – straight – parallel)
Gum width*	Anterior	Smile	Width between the lower edge of the upper lip and the edges of the gum at the front central incisors
Thickness of the upper lip*	Anterior	Smile	the vertical distance from the top edge of the upper lip to the lowest point on the bottom edge of the upper lip
Smile height†	Anterior	Smile	*Medium smile when it reveals 75% to 100% of the upper incisors. *Low smile is less than 75% of upper incisors. *High smile is full length of the incisors and strip of the gums
Maxillary incisor display*	Anterior	Smile	The vertical dimension of the maxillary incisor
Interlabial gap*	Anterior	Smile	The distance between the lower part of the edge of the upper lip to the deepest point on the middle line of the edge of the lower lip
Nasolabial angle††	Lateral	Rest	Measured between the lower nose and the line that intersects the point below the nose and the tangent of the upper lip
Nasolabial angle††	Lateral	Smile	Measured between the minimum nose and the line that intersects the point below the nose and the tangent of the upper lip
Incisor inclination†	Lateral	Smile	Buccolingual positioning of the upper incisor and its relationship to the lip

Each image file of the selected subjects was opened in Photoshop™ software (Photoshop CC 2019, Adobe Systems Inc., California, USA) and adjusted by using the ruler option in the frame, and the dimensions were corrected according to one of the two included rulers in the photographs. The method used to standardize the image was described by Desai et al. [21]. First, the resolution was changed to 300 pixels per inch by going to the “image > image size” option. Then, the ruler function was chosen and set to the millimeter. On the parallel end of the ruler, a 10-mm area close to the smile was measured. This number was divided by 10 and multiplied by the width value found in the image size screen (image > image size). The resulting number was copied and pasted in place of the width reading, and the changes were applied to the JPEG file. In Adobe Photoshop, the previous parameters of the selected subjects were measured and entered into Microsoft Excel® software (Excel 2010, Microsoft Corporation, Washington, USA) for data analysis.

Reliability of the measuring procedure

A total of 45 image files (15 from each group) were randomly selected and remeasured after a four-week interval by the same principal researcher (RMK). The error of the method was analyzed, and paired t-tests were used to assess systematic error between the two measurements.

Statistical analysis

The statistical analysis was performed using SPSS® version 20 software (SPSS, IBM Corporation., Armonk, NY, USA). The one-way ANOVA followed by Tukey’s post-hoc test was used to evaluate the difference between the three groups. Qualitative variables were analyzed using the Chi-square test. A p-value smaller than 0.05 was considered statistically significant.

## Results

Reliability and error of the method

No systematic error was found between the two measuring occasions (p > 0.05; Table 2). The greatest mean value of the method error was 0.553 degrees for the nasolabial angle, and the smallest value was 0.135 mm for the height of the commissure measurements. Method reliability was high, and all error values were smaller than 0.5 mm for the linear measurements and smaller than 1 degree for the angular measurements.

**Table 2:** The reliability of the measuring procedure (error of the method), mean differences, and p-values of significance testing (n = 45). *Error of the method; ^†^Using paired t-test.

Measurement	Filming position	Group	Mean error*	Mean difference	P-value†
Nasolabial angle-rest position	Lateral	Class I	0.432	-0.03	0.108
		Class II division 1	0.161	0.05	0.958
		Class II division 2	0.238	0.07	0.980
Nasolabial angle-smile position	Lateral	Class I	0.412	0.05	0.319
		Class II division 1	0.553	-0.16	0.925
		Class II division 2	0.465	0.13	0.353
Height of the commissure-rest position	Anterior	Class I	0.329	-0.08	0.480
		Class II division 1	0.406	0.04	0.673
		Class II division 2	0.135	0.08	0.144
The thickness of the upper lip-rest position	Anterior	Class I	0.212	-0.02	0.113
		Class II division 1	0.340	-0.06	0.108
		Class II division 2	0.346	0.03	0.135
Lower lip to maxillary incisor-smile position	Anterior	Class I	0.366	0.14	0.961
		Class II division 1	0.386	-0.16	0.983
		Class II division 2	0.227	0.16	0.996
Gum width-smile position	Anterior	Class I	0.439	0.07	0.970
		Class II division 1	0.373	0.04	0.314
		Class II division 2	0.262	-0.09	0.322
The thickness of the upper lip-smile position	Anterior	Class I	0.421	0.10	0.928
		Class II division 1	0.329	-0.11	0.356
		Class II division 2	0.352	0.04	0.483
Maxillary incisor display-smile position	Anterior	Class I	0.355	0.09	0.676
		Class II division 1	0.432	-0.07	0.147
		Class II division 2	0.161	0.08	0.116
Interlabial gap-smile position	Anterior	Class I	0.238	-0.06	0.135
		Class II division 1	0.366	0.11	0.968
		Class II division 2	0.372	0.17	0.981
Incisor inclination-smile position	Lateral	Class I	0.377	0.14	0.955
		Class II division 1			
		Class II division 2			
Smile arc-smile position	Anterior	Class I	0.261	0.12	0.299
		Class II division 1			
		Class II division 2			
Smile height-smile position	Anterior	Class I	0.429	0.04	0.307
		Class II division 1			
		Class II division 2			

Main findings of the linear and angular measurements

The mean values, standard deviations, and p-values of significance testing are presented in Table 3. On the sagittal plane, the nasolabial angle at the rest position had the greatest mean value in the Class I group (106.95 degrees ± 8.79), the smallest mean value in the Class II division 1 group (104.35 degrees ± 10.29), and a moderate mean value in the Class II division 2 group (104.80 degrees ± 5.81). The nasolabial angle at smile had the greatest mean value in the Class I group (103.15 degrees ± 7.86), the smallest mean value in the Class II division 2 group (97.25 degrees ± 10.04), and a moderate mean value in the Class II division 1 group (102.20 degrees ± 13.23). The differences among the malocclusion groups were statistically insignificant for both rest and smile positions (p = 0.589 and p = 0.177, respectively).

**Table 3:** Descriptive statistics of the quantitative measurements (in mm for linear measurements) along with the p-values of significance testing between the three groups. *P < 0.05 (statistically significant); ^†^Using one-way analysis of variance test; ^††^ standard deviation of the variables.

Measurement	Filming position	Group	Mean	Standard deviation††	P-value†
Nasolabial angle-rest position	Lateral	Class I	106.95	8.79	0.589
		Class II division 1	104.35	10.29	
		Class II division 2	104.80	5.81	
Nasolabial angle-smile position	Lateral	Class I	103.15	7.86	0.177
		Class II division 1	102.20	13.23	
		Class II division 2	97.25	10.04	
Height of the commissure-rest position	Anterior	Class I	26.55	4.12	0.001*
		Class II division 1	31.27	4.26	
		Class II division 2	25.23	5.79	
The thickness of the upper lip-rest position	Anterior	Class I	6.25	1.33	<0.001*
		Class II division 1	8.77	1.69	
		Class II division 2	6.25	1.33	
Lower lip to maxillary incisor-smile position	Anterior	Class I	0.00	0.00	1.000
		Class II division 1	0.00	0.00	
		Class II division 2	0.00	0.00	
Gum width-smile position	Anterior	Class I	0.25	0.72	0.188
		Class II division 1	0.50	1.10	
		Class II division 2	0.05	0.22	
The thickness of the upper lip-smile position	Anterior	Class I	5.60	1.50	0.647
		Class II division 1	6.28	2.99	
		Class II division 2	5.89	2.16	
Maxillary incisor display-smile position	Anterior	Class I	8.25	2.05	0.064
		Class II division 1	10.08	3.79	
		Class II division 2	8.09	2.60	
Interlabial gap-smile position	Anterior	Class I	11.20	2.91	0.061
		Class II division 1	12.68	5.03	
		Class II division 2	9.92	2.27	

In the anterior filming positioning, the thickness of the upper lip at rest position was the greatest in the Class II division 1 group (8.77 mm ± 1.69) and the same mean value in both Class I and Class II division 2 groups (6.25 mm ± 1.33) with statistically significant differences (p < 0.001). The thickness of the upper lip at the smile position was also the greatest in the Class II division 1 group (6.28 mm ± 2.99), the smallest in the Class I group (5.60 mm ± 1.50), and had a moderate mean value in Class II division 2 group (5.89 mm ± 2.16) with no statistically significant differences (p = 0.647). Maxillary incisor display measurement demonstrated the greatest mean value in the Class II division 1 group (10.08 mm ± 3.79), the smallest mean value in the Class II division 2 group (8.09 mm ± 2.60), and a moderate mean value in the Class I group (8.25 mm ± 2.05) with no statistically significant intergroup difference (p = 0.064). Interlabial gap measurement also showed the greatest mean value in the Class II division 1 group (12.68 mm ± 5.03), the smallest mean value in the Class II division 2 group (9.92 mm ± 2.27), and a moderate mean value in the Class I group (11.20 mm ± 2.91) with no statistically significant intergroup difference (p = 0.061).

Incisor inclination, smile arc, and smile height

Incisor Inclination

In the Class I group, most patients (80%) had normally inclined incisors, a few patients (15%) had retroclined incisors, and the other patients (15%) had proclined incisors. In the Class II division 1 group, most patients (85%) had proclined incisors, 15% had normally inclined incisors, and no patients (0%) had retroclined incisors. All patients (100%) in the Class II division 2 group had retroclined incisors. The difference between the three groups was statistically significant for the incisor inclination scale (p = <0.001; Table 4).

**Table 4:** Descriptive statistics of the categorical variables and the p-values of significance testing. *Statistically significant; ^†^Using the Chi-square test.

Measurement	Scale of measurement	Group	Absolute distribution	Relative distribution (%)	P-value†
Incisor inclination	Normally inclined	Class I	16	80	<0.001*
		Class II division 1	3	15	
		Class II division 2	0	0	
	Retroclined	Class I	1	5	
		Class II division 1	0	0	
		Class II division 2	20	100	
	Proclined	Class I	3	15	
		Class II division 1	17	85	
		Class II division 2	0	0	
Smile arc	Reverse	Class I	0	0	<0.001*
		Class II division 1	9	45	
		Class II division 2	0	0	
	Straight	Class I	20	100	
		Class II division 1	0	0	
		Class II division 2	0	0	
	Parallel	Class I	0	0	
		Class II division 1	11	55	
		Class II division 2	20	100	
Smile height	Medium	Class I	17	85	0.887
		Class II division 1	16	80	
		Class II division 2	17	85	
	High	Class I	3	15	
		Class II division 1	4	20	
		Class II division 2	3	15	

Smile Arc

All patients (100%) in the Class I group had a straight smile arc. In the Class II division 1 group, more than half of the patients (55%) had a parallel smile arc, and 45% of patients had a reverse smile arc. On the other hand, all patients (100%) in the Class II division 2 group had a parallel smile arc. The difference between the three groups was statistically significant for the smile arc scale (p < 0.001).

Smile Height

In both Class I and Class II division 2 groups, 85% of patients had a medium smile height, and 15% had a high smile height. In contrast, 80% of patients in the Class II division 1 group had a medium smile height, and the remaining proportion (20%) had a high smile height. However, no significant intergroup differences were detected for this variable (p = 0.887).

## Discussion

The effect of skeletal Class II malocclusion on smile characteristics was evaluated in this study, which included 60 patients with an age range between 18 and 28 years. This study appears to be the first to compare the effect of skeletal Class II malocclusion with its two divisions on the dynamics of the smile in comparison with skeletal Class I malocclusion using still images acquired from video recordings.

In this study, the value of the nasolabial angle was smaller in Class II division 1 and 2 groups than in the Class I group. The upper lip and incisor protrusion can explain this in Class II malocclusion patients. This result agrees, in general, with that of a previous study [22]. The commissure height was measured at rest position and was the smallest in the Class II division 2 group and the greatest in the Class II division 1 group, with statistically significant differences. This can be explained by the increase in the vertical dimension of the maxilla in Class II division 1 malocclusion patients and the decrease in the lower facial third in Class II division 2 malocclusion patients.

The thickness of the upper lip was noticed to be the greatest in the Class II division 1 group and similar values in the other groups with statistically significant differences at the rest position and no statistically significant differences at the smile position. These findings disagree with those of Alkhalaf et al. [15], whose sample included dental no skeletal Class II malocclusion patients. Moreover, gender was considered in that study, and the soft-tissue thickness differs between males and females. The value of gum width was the greatest in the Class II division 1 group and the smallest in the Class II division 2 group without statistically significant differences. These results may be due to the increase in the vertical dimension of the maxilla in Class II division 1 malocclusion patients. Another possible cause is the hyperactivity of the levator muscle of the upper lip, which leads to more gum display in such patients. These results agree in general with those of previous studies [19,23-25].

For maxillary incisor display, the results were the greatest in the Class II division 1 group and the smallest in the Class II division 2 group without statistically significant differences. These results can be explained by the increase in the vertical dimension of the maxilla in Class II division 1 malocclusion patients, which leads to more incisor display at the smile position, and another reason is the decrease in the lower facial third in Class II division 2 malocclusion patients leading to make the upper line of the lower lip higher than the upper incisal edge and therefore less incisor display. Hence, the present findings corroborate the results of Kim and Freitas [23] but disagree with those of Alkhalaf et al. [15] and Sabri [26].

The value of the interlabial gap was the greatest in the Class II division 1 group and the smallest in the Class II division 2 group without statistically significant differences. This can be caused by the increase in the frontal height of the maxilla in Class II division 1 malocclusion patients leading to labial inefficiency at the rest position and increased interlabial gap at the smile position. These findings agree with those of previous studies [14,27].

Incisor inclination revealed that most Class I malocclusion patients had normally inclined incisors, most Class II division 1 malocclusion patients had proclined incisors, and all Class II division 2 malocclusion patients had retroclined incisors. These can be demonstrated by the effect of lower lip efficacy on upper incisor positions. The present findings corroborate, in general, the results of Nanda [19].

Smile arc distribution was statistically significant as a straight arc in all Class I malocclusion patients, reverse arc in nearly half of Class II division 1 malocclusion patients, and parallel arc in all Class II division 2 malocclusion patients the other patients in Class II division 1 group. These findings agree with those of previous studies by Tjan [28] and Dong et al. [13]. For smile height, most of the patients in the three groups had medium smile height, and the others had a high smile height without significant differences. These results agree with those of Islam et al. [29].

Limitations

Although the patient was trained several times to smile naturally before video recording, this natural smile position may have been affected slightly by any possible distraction when the patient was asked to hold the rulers. Another method of calibration of the images should be thought of for more reproducible facial expressions. This study evaluated the natural smile, and the analysis could have included other types of smile, such as the posed smile and the maximum smile. This study had a relatively small sample size and did not consider age and gender. Therefore, there is a need for more studies with larger samples to evaluate the influence of different types of malocclusion on smile characteristics considering age and gender.

## Conclusions

Skeletal malocclusion affects the characteristics and dynamics of the smile in the anterior and lateral imaging angles. Gum width, maxillary incisor display, and the interlabial gap at the smile position were greater in patients with Class II division 1 in the anterior filming shots. There was no difference in the amount of coverage of the lower lip of the upper incisors between the studied groups. The normal inclination of the upper incisors was evident in patients with Class I malocclusion, while the proclination was evident in patients with Class II division 1 malocclusion, and the retroclination was found in patients with Class II division 2 malocclusion in the anterior imaging angle at the smile position. The orthodontist should always analyze patients’ facial expressions, including those related to the upper and lower lips at rest and when patients smile naturally. Accordingly, treatment planning could be built to improve the characteristics of the natural smile in patients with Class I and Class II malocclusions.
